# New Oral Anticoagulants in Acute Coronary Syndrome: Is There Any Advantage Over Existing Treatments?

**Published:** 2014-09-01

**Authors:** Andrea Messori, Valeria Fadda, Roberta Gatto, Dario Maratea, Sabrina Trippoli

**Affiliations:** 1 HTA Unit, ESTAV Toscana Centro, Regional Health Service, 50100 Firenze, Italy

**Keywords:** Anticoagulants, Acute Coronary Syndrome, Rivaroxaban, Ticagrelor

## Abstract

**Background:**

After an acute coronary syndrome, dual antiplatelet therapy with clopidogrel plus aspirin is still a standard of care, but several new approaches have been investigated.

**Objectives:**

The present study re-examined the studies published thus far on this topic to evaluate the effectiveness of dual antiplatelet therapy in comparison to some of these new approaches (mainly, ticagrelor + aspirin and dual therapy plus a new oral anticoagulant [NOAC]; i.e., “triple therapy”).

**Materials and Methods:**

The clinical material was directly derived from that reported in recent meta-analyses. Our re-analysis relied on standard equivalence methods in which interpretation is based on Relative Risks (RRs) along with their 95% Confidence Intervals (CI). The equivalence margins employed in our statistical testing were directly derived from those reported in randomized studies.

**Results:**

The equivalence margins were initially set at RR ranging from 0.775 to 1.29. According to these margins, triple therapy based on any NOAC proved to be superior to dual therapy alone, but at the same time demonstrated its equivalence with dual therapy. The results for apixaban-based triple therapy were inconclusive (not superior, not not-inferior, not equivalent and, of course, not inferior to the controls). Those for rivaroxaban-based triple therapy showed that this combination treatment was superior to dual therapy alone and failed to meet the criterion of equivalence. In the comparison between rivaroxaban-based triple therapy and ticagrelor + aspirin, the RR was 1 and its 95% CI remained within a post-hoc margin of ± 15%.

**Conclusions:**

Even if one considers the most effective NOAC in combination with clopidogrel + ticagrelor, this triple therapy is not more effective than ticagrelor + aspirin. On the other hand, the increased risk of bleeding with triple regimens is well demonstrated. We therefore conclude that these triple regimens did not play any important roles in the patients experiencing an acute coronary syndrome.

## 1. Background

After an acute coronary syndrome, dual antiplatelet therapy with clopidogrel plus aspirin is still considered a standard of care (1). However, new therapeutic approaches are increasingly being explored (2, 3) and, in particular, addition of a new oral anticoagulant (NOAC) to dual therapy (“triple therapy”) has been studied quite extensively.

With regard to this form of triple therapy, one recent systematic review (4) has pointed out that adding a NOAC to dual therapy determines a reduced incidence of major adverse cardiovascular events (MACEs; defined as the composite of all-cause mortality, myocardial infarction, or stroke) with a Relative Risk (RR) of 0.87 (95% Confidence Interval [CI], 0.80 - 0.95). On the other hand, there is an increased risk of bleeding (RR = 2.34; 95% CI, 2.06 - 2.66).

The network meta-analysis by Gatto et al. (5) evaluated two direct comparisons (rivaroxaban + clopidogrel + aspirin vs. clopidogrel + aspirin and ticagrelor + aspirin vs. clopidogrel + aspirin) based on “real” head-to-head trials and one indirect comparison (rivaroxaban + clopidogrel + aspirin vs. ticagrelor + aspirin) that was handled through the network analysis. This study confirmed that both rivaroxaban + clopidogrel + aspirin and ticagrelor + aspirin were superior to clopidogrel + aspirin (end-point = MACE). Moreover, an RR of 1.00 (95%CI, 0.87 - 1.15) was estimated for the indirect comparison of rivaroxaban + clopidogrel + aspirin to ticagrelor + aspirin (5).

Pre-defined superiority margins (6) are commonly used in randomized controlled trials to identify a threshold between clinically relevant benefits and irrelevant ones and to consequently carry out power calculations. In the ATLAS-ACS-2 TIMI 51 trial (7) that compared rivaroxaban + clopidogrel + aspirin to clopidogrel + aspirin (primary end-point = MACE), the superiority margin was expressed as a 22.5% RR reduction. This superiority margin can also be assumed to represent the equivalence margin in terms of RR (with values ranging from 0.775 to 1.29, where 1.29 = 1/0.775).

To better interpret the effectiveness data discussed above, in the present analysis, we carried out an equivalence test (6) in which we combined the RRs (end-point = MACE) found by Oldgren et al. for dual therapy plus NOAC vs. dual therapy alone (4) with the margins adopted in the ATLAS-ACS-2 TIMI 51 trial (7). In particular, we interpreted the available evidence by comparing the incremental benefits estimated for various treatments against a threshold benefit representing the margin of therapeutic equivalence (6).

## 2. Materials and Methods

The present study was a re-analysis of published material in which we introduced a series of original equivalence tests aimed at comparatively evaluating the effectiveness of the treatments under examination. The therapeutic issue considered herein was treatment of patients after an acute coronary syndrome and the objective was to carry out a comparative assessment of various therapeutic options. The treatments included in these comparisons were as follows: any NOAC + clopidogrel + aspirin, apixaban + clopidogrel + aspirin, rivaroxaban + clopidogrel + aspirin, clopidogrel + aspirin, and ticagrelor + aspirin. The same primary end-point (MACE) was evaluated across all these treatments.

The equivalence tests were carried out on the basis of the comparison of 95% CI of RRs as indicated by Ahn et al. (6). The margins employed for our equivalence tests were directly obtained from the power calculations reported in the original randomized trials. In this framework, the superiority margins employed in the randomized trials were assumed to represent, at the same time, the margins of therapeutic equivalence (8). Since all results were reported as RRs with 95% CIs, when necessary, the lower margin of the 95% CI was estimated from the reciprocal of the upper margin, or vice-versa. The results of the equivalence tests were interpreted according to the standard criteria (6).

## 3. Results

**Figure 1.**
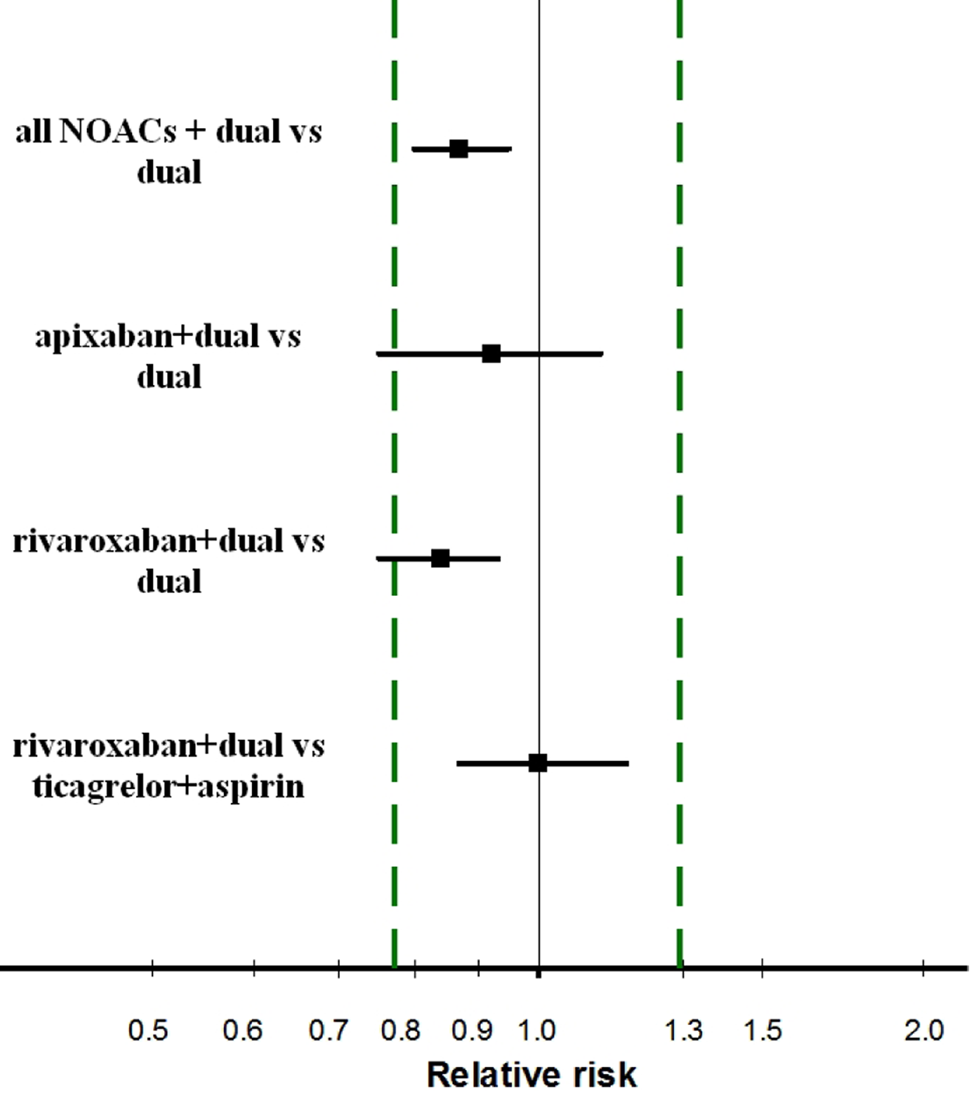
Rates of MACE after Acute Coronary Syndrome in the Patients Treated with Different Combinations of NAOCs and/or Antiplatelet Agents The equivalence test is based on the area comprised between the two vertical dashed lines reflecting the pre-determined equivalence margins (from 0.775 to around 1.29). Each horizontal bar indicates two-sided 95% CI for the RR (solid square). The criterion for demonstrating equivalence is when both extremes of the 95% CI remain within the two vertical lines. Comparisons (top to bottom): 1) triple therapy with clopidogrel + aspirin + any NOAC vs. dual therapy; 2) triple therapy with apixaban + clopidogrel + aspirin vs. dual therapy (APPRAISE-2 trial); 3) triple therapy with rivaroxaban + clopidogrel + aspirin vs. dual therapy (ATLAS-2 trial); 4) triple therapy with rivaroxaban + clopidogrel + aspirin vs. ticagrelor + aspirin. All the effectiveness data were derived from Olsgren’s meta-analysis (4) with the exception of the fourth comparison the data of which were obtained from Gatto et al. (5).

The present study results have been summarized in Figure 1. Considering the pooled RR for all NOACs, triple therapy proved to be superior to dual therapy alone, but at the same time demonstrated its equivalence with dual therapy (according to the equivalence margin of around ± 22.5%). This contradiction can, in general, have two different explanations: too wide equivalence margins or very small magnitude of the benefit despite its statistical significance. In this case, the first explanation is preferable (6).

Figure 1 shows also the equivalence analyses focused on either apixaban or rivaroxaban given as triple therapy in comparison to dual therapy. The results for apixaban were inconclusive (not superior, not not-inferior, not equivalent, and, of course, not inferior to the controls), but those for rivaroxaban were more informative. In fact, this NOAC, when combined at the above dosages with clopidogrel + aspirin, was superior to dual therapy alone and, accordingly, failed to meet the criterion of equivalence.

Overall, our comparative overview of the available evidence confirms the choice made by Gatto et al. (5) to select rivaroxaban as the most effective NOAC given as triple therapy and to consequently compare rivaroxaban-based triple therapy to ticagrelor + aspirin using an indirect network meta-analysis. Interestingly enough, this indirect comparison not only showed that there was no difference in effectiveness between these two treatments, but also showed that the 95% CI for this comparison (from 0.87 to 1.15) differed from unity by not more than 15 percentage points and, consequently, met a very reasonable post-hoc criterion of equivalence of ± 15%.

## 4. Discussion

Given that ticagrelor + aspirin is equivalent to rivaroxaban + clopidogrel + aspirin in terms of effectiveness, the role of this latter triple combination seems to be questionable. There are at least three reasons that prevent recognition of an important role for this triple treatment:

- according to the present analysis, there is the proof of no improved effectiveness for the triple therapy (which is more informative than no proof of increased effectiveness),
- there is an increased risk of bleeding for triple therapy according to the findings of Olgren et al. (4), and
- as pointed out by Verheugt (9), "three could be a crowd".

In fact, the present analysis showed that even if one considers the most effective NOAC in combination with clopidogrel + ticagrelor, this triple therapy is not more effective than ticagrelor + aspirin. On the other hand, the increased risk of bleeding with triple regimens is so clear-cut (4) that a more specific statistics on this point is unnecessary.

We therefore concluded that these triple regimens did not play any important roles in the patients experiencing an acute coronary syndrome. Further studies and further analyses could however be warranted to better define the therapeutic role of dual treatments including one NOAC plus aspirin.
